# Genome-Wide Association Study of Grain Architecture in Wild Wheat *Aegilops tauschii*

**DOI:** 10.3389/fpls.2017.00886

**Published:** 2017-05-31

**Authors:** Sanu Arora, Narinder Singh, Satinder Kaur, Navtej S. Bains, Cristobal Uauy, Jesse Poland, Parveen Chhuneja

**Affiliations:** ^1^School of Agricultural Biotechnology, Punjab Agricultural UniversityLudhiana, India; ^2^Crop Genetics, John Innes CentreNorwich, United Kingdom; ^3^Wheat Genetics Resource Center, Department of Plant Pathology, Kansas State University, ManhattanKS, United States; ^4^Department of Plant Breeding and Genetics, Punjab Agricultural UniversityLudhiana, India

**Keywords:** *Aegilops tauschii*, genetic diversity, genome-wide association mapping, genotyping by sequencing, grain size, single nucleotide polymorphic markers

## Abstract

*Aegilops tauschii*, the D-genome progenitor of *Triticum aestivum*, encompasses huge diversity for various traits of potential economic importance such as yield, biotic and abiotic stress tolerance, quality and nutrition. In the present study, variation for grain size in *Ae. tauschii* germplasm was studied and its genetic basis dissected using genome-wide association study (GWAS). Grain length, width, and weight evaluated in 177 *Ae. tauschii* accessions over 3 years showed near normal distribution with 1.74-, 1.75-, and 2.82-fold variation, respectively. These lines were genetically characterized using genotyping-by-sequencing (GBS) protocol that produced 11,489 single nucleotide polymorphic (SNP) markers. Genetic diversity analysis revealed the presence of two distinct subgroups (designated as lineage 1 and 2) in *Ae. tauschii*. Based on GBS markers, the genetic similarity was calculated between the accessions and GWAS was conducted using 114 non-redundant accessions and 5,249 SNP markers. A total of 17 SNPs associated with grain size traits distributed over all the seven chromosomes were revealed with 6D, 5D, and 2D harboring most significant marker–trait associations. Some of the chromosomal regions such as 6D_66.4–71.1 cM, 1D_143.5–156.7 cM, and 2D_89.9–92.5 cM had associations with multiple traits. Candidate genes associated with cell division and differentiation were identified for some of the associated SNP markers. Further efforts to validate these loci will help to understand their role in determining grain size and allelic diversity in current germplasm and its effect on grain size upon transfer to bread wheat background.

## Introduction

Human population has doubled during the last 45 years and is expected to reach 9.7 billion people by 2050 (United Nations, Department of Economic and Social Affairs, Population Division, 2015). Feeding such a burgeoning population would require raising the overall food production by 70% (FAO, 2009). However, the present rate of increase in the production of three major cereals may not keep pace with the growing world population. Improving crop yield is an essential solution to solve world’s hunger issue. Grain yield is a complex trait and it represents the culmination of diverse array of vegetative and reproductive processes and their interplay with environmental factors (Quarrie et al., 2006). Grain yield is determined by two components; number of grains per m^2^ and grain weight. Modern breeding has improved yield by increasing grains per m^2^ due to the utilization of dwarfing genes (Rht). Semi-dwarf wheat varieties led to increase in harvest index and were the main contributors of the Green Revolution in 1960s and 1970s (Bonneau et al., 2013). Also, the genes affecting flowering time and photoperiod response (*Vrn* and *Ppd*) helped to improve adaptation of plant phenology to various environmental factors leading to an improvement of growth and development. However, for further improvement of yield potential, grain weight is an important component to target. Grain weight in turn is determined by grain length, width, and area, which are inherited in a stable manner and show higher heritability than overall yield (Kuchel et al., 2007).

Wheat is a natural allohexaploid with A, B, and D genomes contributed by *Triticum urartu*, a close relative of *Aegilops speltoides* and *Aegilops tauschii* (syn. *A. squarrosa, Tritcum tauschii*), respectively (Petersen et al., 2006). Only a few accessions of the *Ae. tauschii* were involved in these rare hybridizations leading to limited D-genome variation represented in hexaploid bread wheat (Cox, 1997). On the other hand, a lot of genetic variability is available in the D-genome of *Ae. tauschii* germplasm for various traits of agronomic importance such as high and low molecular weight glutenin subunits (Gianibelli et al., 2002; Yan et al., 2003a), gliadins (Yan et al., 2003b) and amplified fragment length polymorphism (AFLP) and simple sequence repeat (SSR) markers (Pestsova et al., 2000; Naghavi et al., 2007). The nucleotide sequence diversity has been estimated to be 30-fold higher in *Ae. tauschii* than in the D-genome of *Triticum aestivum* (Caldwell et al., 2004).

To increase D-genome diversity in bread wheat, in a concerted effort spanning 25 years, the International Center for Maize and Wheat Improvement (CIMMYT) has generated more than 1,200 synthetic hexaploid wheat lines by crossing elite tetraploid durum with *Ae. tauschii* accessions (Trethowan and Mujeeb-Kazi, 2008). *Ae. tauschii* has also been used to introgress various traits of economic importance such as biotic stress resistance (Miranda et al., 2006; Olson et al., 2013) and yield traits (Watanabe et al., 2006) into bread wheat. However, developing synthetic wheats and introgressing target traits from *Ae. tauschii* to cultivated wheats is a very time-consuming and labor-intensive, while the utility of the introgressed alleles becomes apparent only after transfer. Study of the allelic effects and identification of the linked markers in the *Ae. tauschii* background can enable targeted introgressions to be made.

Recent advances in genomic technologies has enabled a better understanding of the genetic basis of the variation in large germplasm sets using genome-wide association studies (GWAS). GWAS is one such approach that can be used for identification and high resolution mapping of useful genetic variability from germplasm sets that have resulted from many rounds of historical recombination (Yu and Buckler, 2006). Marker–trait associations (MTAs) result from linkage disequilibrium (LD) between assayed markers and the causal genes. However, to get the associations at a fine mapping resolution, GWAS requires screening the genome with large number of markers. Though this has been a challenge for wild-relatives and understudied species, due to rapid developments in the next generation sequencing, genotyping has become more cost effective enabling the use of large number of markers covering the whole genome. Genotyping-by-sequencing (GBS) is a restriction enzyme-based complexity reduction (representing small fraction of the entire genome) approach that involves sequencing of multiplexed samples coupled with genome-wide molecular marker discovery and genotyping. GBS was originally developed by Elshire et al. (2011) for high resolution association studies in maize. A two enzyme GBS approach has been developed by Poland et al. (2012) and has been applied in major field crops since then.

Most studies in *Ae. tauschii* germplasm have focused on understanding the genetic and morphological diversity of this species. Limited studies have utilized GWAS in *Ae. tauschii* for traits of economic importance including those on cadmium stress (Qin et al., 2015), P-deficiency (Liu et al., 2015a) and some morphological traits (Liu et al., 2015b). The present investigation was undertaken to understand the genetic diversity in *Ae. tauschii* germplasm and to perform genome-wide association studies for grain length, width and weight using GBS-based single nucleotide polymorphic (SNP) markers.

## Materials and Methods

### Plant Material

Plant material comprised of 177 *Ae. tauschii* accessions maintained at the Wheat Germplasm Collection, Punjab Agricultural University (PAU), Ludhiana, India (30°53′54.2″N 75°48′33.9″E). These accessions were originally obtained from the Wheat Genetics Resource Center (Kansas State University), University of Missouri, CIMMYT, International Center for Agricultural Research in the Dry Areas (ICARDA), and The Leibniz Institute of Plant Genetics and Crop Plant Research (IPK) Gatersleben. The detailed information of these accessions is provided in the **Supplementary Table S1**.

### Phenotyping

The *Ae. tauschii* accessions were grown at Punjab Agricultural University (PAU) for three consecutive seasons (2011–2013). Each accession was planted in a single row of 2 m length with 0.7 m spacing between the rows. The spikes were harvested at physiological maturity. The seeds were manually removed from the spikelet as they were adhered to the lemma and palea of the glume. 50-grain weight (50-GWT), grain length (GLN), and grain width (GWD) was recorded for each accession during the three cropping seasons. The measurements for grain length and width were taken using a digital Vernier caliper and any bias associated with manual measurements was reduced by using average value of 10 grains for each accession. The 50-grain weight was determined using electronic balance in three replications for each accession.

### Statistical Analysis

Descriptive analysis, ANOVA, correlation analysis and heritability estimates were conducted for the 3 years data in the R statistical package (R Core Team, 2013). Variation among genotypes for all phenotypic traits were studied using mean, range, standard deviation (SD), and coefficient of variation. Variance components for all traits were analyzed using general linear model to detect the effect of genotypes, year, and genotypes × year interaction. Pearson’s correlation coefficients for grain length, width, and weight were calculated based on individual year’s data to assess correlations between these traits as well as same trait over the years. Best linear unbiased predictors (BLUP) were estimated for each line and each trait using lme4 package in R. The broad-sense heritability for each trait was estimated by the formula *H*^2^ = VG/(VG + VE) where VG and VE represent estimates of genetic and environmental variance, respectively.

### Genotyping and SNP Discovery

Genomic DNA was extracted from fresh young leaves using the cetyltrimethylammonium bromide (CTAB) method. DNA was quantified using PicoGreen and concentrations were normalized to 20 ng/μl. The GBS libraries were constructed in 96-plex following co-digestion with two restriction enzymes *Pst*I (CTGCAG) and *Msp*I (CCGG) followed by barcoded adaptors ligation of individual samples. The samples were pooled per plate and PCR amplified (Poland et al., 2012). Each library was sequenced on the Illumina HiSeq 2000 platform using single end sequencing from *Pst*1 sites. The raw Illumina reads were assigned to their respective samples on exact match to barcode sequence and trimmed to 64 bp. The unique tags were internally aligned to identify putative SNPs by allowing 1–3 bp mismatch in 64 bp sequence using a custom Java script in TASSEL 3. To identify the true SNP markers, population-based SNP calling approach was used (Elshire et al., 2011). The Fisher exact test was used to determine if the two alleles were independent SNP markers. The SNP markers with minor allele frequency (MAF) less than 5% and missing data greater than 30% were removed from the analysis. The remaining SNP markers were positioned on the D-genome of the Synthetic × Opata reference genome map (Chapman et al., 2015; Edae et al., 2015).

### Genetic Diversity and Population Structure Analysis

The missing values for SNP markers were imputed by “mean” method using “rrBLUP” package available in R. The resulting matrix was used for calculating the eigenvalues to make principal component analysis (PCA) plot. The genetic structure was analyzed using Bayesian inference program STRUCTURE 2.3.4. using a set of 10,000 polymorphic SNP markers. The genotypes were treated as an admixture population and used along with the allele frequencies correlated model. A total of 10,000 burn-in iterations followed by 100,000 Markov chain monte carlo (MCMC) iterations for *K* = 1–8 clusters were used to identify the optimal cluster (K). For each *K*, five independent runs were produced. Based on the final *K* value, the population was divided into two lineages. The genetic differentiation among the lineages was further investigated by pairwise fixation index (F_ST_) using the software DnaSP 5.10.01 with 1,000 permutations. The evolutionary relationship among accessions was determined by constructing a neighbor-joining (NJ) tree with 1,000 bootstrap iterations using the nucleotide p-distance model under pairwise deletion in MEGA 6.0.

### Linkage Disequilibrium and Association Analysis

Based on GBS-SNP markers, a triangular identity-by-state genetic similarity matrix was obtained for all possible pairs of accessions using a custom R script to find accessions with high genetic similarity. The accessions in each identical group were analyzed for phenotypic diversity for days to flowering, stripe rust reaction, growth habitats, spike morphology, and origin. Based on both genetic and phenotypic identity, duplicate accessions were removed from further analysis. Overall, 36% of the accessions were not included in the GWAS due to very high genetic similarities.

For GWAS analysis, no SNP imputation was performed. Only those SNPs were used that successfully mapped to the D-genome in Synthetic × Opata reference genome with minor allele frequency (MAF) ≥ 5% and missing data less than 30%. LD estimates between marker pairs were obtained using TASSEL v5 with 2,424 and 1,446 SNPs for lineage 1 and 2, respectively. The pairwise LD values (*r*^2^) were plotted against genetic distance, a locally weighted polynomial regression (LOESS) curve was fitted using statistical program R, and the pattern of LD decay was determined as the genetic distance where LOESS curve intercepts the *r*^2^ threshold of 0.1.

MTAs of 5,249 SNP markers with MAF > 0.05 were evaluated on the BLUP values for 114 non-redundant accessions. GWAS was conducted using a recently developed model selection algorithm, the Fixed and random model Circulating Probability Unification (FarmCPU; Liu et al., 2016). The algorithm takes into account the confounding problem between covariates and test marker by using both Fixed Effect Model (FEM) and a Random Effect Model (REM). The first three principal components calculated using GAPIT (Lipka et al., 2012) were used as covariates. The default *p*-value threshold that FarmCPU uses is Bonferroni-corrected threshold with 0.01. As Bonferroni-corrected threshold is overly strict when the LD among genotypic markers is large, so the threshold was calculated using the parameter “p.threshold = 0.05/number of markers” using 1,000 permutations. In this function, the phenotypes are permuted to break the relationship with the genotypes. A vector of minimum *p*-value of each experiment is output and the 95% quantile value of the vector is recommended for p.threshold in FarmCPU model. The threshold calculated by FarmCPU for the given traits is 4.68 and was used as a cut-off to define MTAs. The quantile–quantile (Q–Q) plot was used for assessing how fit the model was to account for population structure. For the significantly associated markers, the allelic effects were determined by representing phenotype data for alleles as box-plots and using Kruskal–Wallis test to find whether the alleles differ significantly for the associated traits.

### Putative Candidate Gene Analysis

To find candidate genes or related proteins for the reported MTAs, we performed a BLASTn search of the significantly associated GBS tags against the TGACv1 chromosome sequences (International Wheat Genome Sequencing Consortium, 2014)^1^ and the position where the tag hit the best match was extended by 10 kb in both directions. The sequence was used to BLAST search Ensembl *T. aestivum* database to find predicted genes or proteins within this region. The proteins were predicted for all the sequences using FGENESH and any candidate protein or domain was searched using BLASTp in NCBI^2^.

## Results

### Phenotypic Evaluation

**FIGURE 1:**
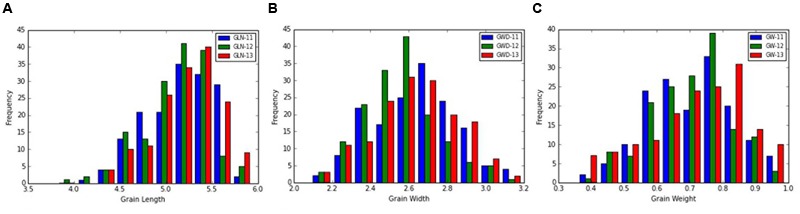
Phenotypic distribution for grain **(A)** length, **(B)** width, and **(C)** weight in the year 2011, 2012, and 2013.

The frequency distribution and descriptive statistics of grain length, width and 50-grain weight revealed a wide variation in *Ae. tauschii* (**Figure 1**). The variation ranged from 1.80 to 3.15 mm (mean ± SD = 2.58 ± 0.29 mm) for grain width, 3.61 to 6.30 mm (5.14 ± 0.38 mm) for grain length, and 0.35 to 0.99 mg (0.69 ± 0.14 mg) for grain weight (**Table 1**). Overall, 1.74-, 1.75-, and 2.83-fold variation for grain length, width, and weight was recorded in this panel of *Ae. tauschii* accessions.

**Table 1:** Descriptive statistics, broad sense heritability (*H*^2^) and *F*-value from analysis of variance for the grain size descriptors in year 2011, 2012, and 2013.

Trait	Year	Mean ± SD	CV%	Range		*H*^2^	*F*-values from ANOVA	
				Min	Max		Year	Genotype
Grain length (mm)	2011	5.15 ± 0.38	7.30	4.19	6.30	0.57	10.5^∗∗∗^	6.45^∗∗∗^
	2012	5.08 ± 0.39	7.67	3.61	6.09			
	2013	5.19 ± 0.37	7.12	3.90	6.25			
	2011–2013	5.14 ± 0.38	7.43	3.61	6.30			
Grain width (mm)	2011	2.61 ± 0.30	11.4	1.95	3.15	0.41	22.6^∗∗∗^	10.8^∗∗∗^
	2012	2.53 ± 0.21	8.30	1.80	3.12			
	2013	2.60 ± 0.24	9.20	1.85	3.14			
	2011–2013	2.58 ± 0.29	8.88	1.80	3.15			
Grain weight (mg)	2011	0.700 ± 0.13	18.6	0.418	0.962	0.74	1.47^ns^	15.8^∗∗∗^
	2012	0.694 ± 0.12	17.2	0.405	0.997			
	2013	0.704 ± 0.16	22.7	0.350	0.987			
	2011–2013	0.69 ± 0.14	20.3	0.35	0.99			

*ns, not significant; ^∗∗∗^, ^∗∗^, and ^∗^, significant at *p* < 0.001, *p* < 0.01, and *p* < 0.05, respectively.*

**FIGURE 2:**
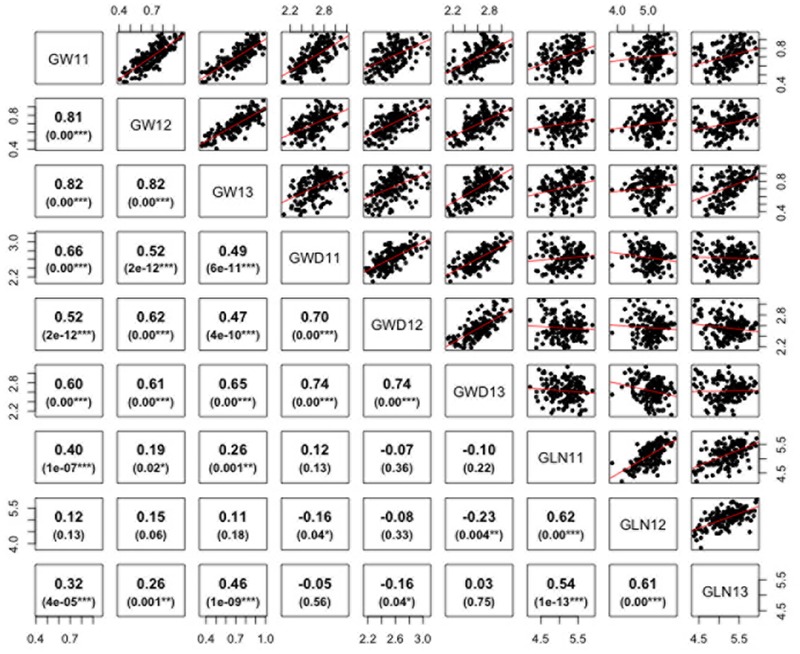
Correlations between grain weight, length, and width in *Ae. tauschii* for 2011–2013. *p*-Value for significant correlations is shown at the bottom. ^∗∗∗^, ^∗∗^, and ^∗^, Significant at *p* < 0.001, *p* < 0.01, and *p* < 0.05, respectively.

ANOVA analysis revealed significant differences among genotypes for the three traits defining grain dimensions. A strong positive correlation was observed between years for grain weight (*r* = 0.80–0.82), grain width (*r* = 0.70–0.74) and grain length (*r* = 0.62–0.54). The correlation coefficients illustrated significant positive correspondence of grain weight with both grain width (*r* = 0.47–0.66) and length (*r* = 0.11–0.46), however, no significant correlation was found between grain length and width (*r* = -0.23–0.12) (**Figure 2**). Broad sense heritability estimates ranged from 0.41 for grain width to 0.74 for grain weight. The distribution of BLUP values, calculated from phenotypic data to obtain unbiased mean estimates, for grain length, width, and weight is presented in Supplementary Figure S1.

### GBS-SNP Markers Statistics

Initially, a total of 59,830 putative SNP markers were called for the 177 *Ae. tauschii* accessions using a SNP calling pipeline implemented in TASSEL 3. These were reduced to 24,547 SNP markers, after removing the SNPs with MAF < 0.05 and missing data ≥30% which led to almost 59% culling. These marker tags were integrated into the anchored assembly from POPSEQ based Synthetic × Opata reference genetic map (Edae et al., 2015). After discarding markers with low alignment score and that mapped to the A and B genomes, we found map positions for 11,489 markers (46.8%). Chromosomes 1D–7D contained 1,672, 1,465, 2,598, 754, 1,156, 1,247, and 1,066 SNP markers, respectively.

### Molecular Diversity and Structure Analysis

**FIGURE 3:**
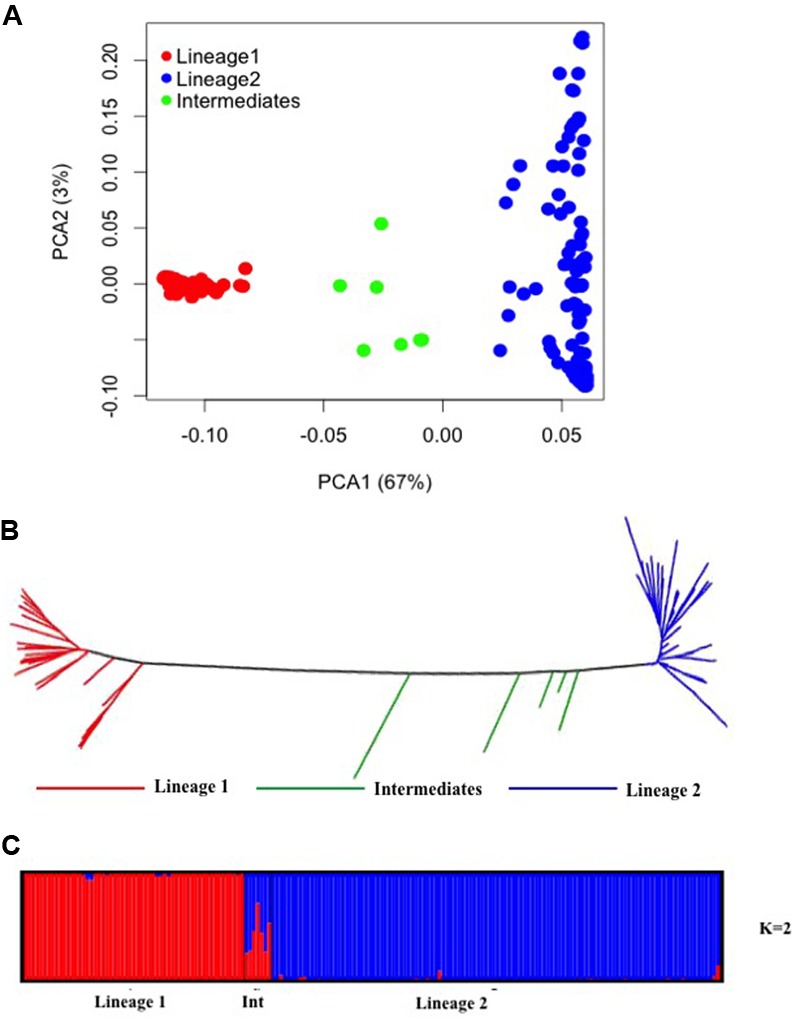
Diversity analysis of *Aegilops tauschii* accessions based on GBS makers. **(A)** Principal component analysis. **(B)** Phylogenetic neighbor-joining tree. **(C)** Structure results at *K* = 2.

PCA of 24,547 SNPs was used to assess the clustering of genetic variation in 177 *Ae. tauschii* accessions. The first two PCA components explained 70% of the total variation with 67% for PC1 and 3% for PC2 (**Figure 3A**). PC1 grouped the accessions into two major clusters, referred to as lineage 1 and lineage 2, with seven accessions located between the two lineages. Lineage 1 comprised almost exclusively of accessions from subspecies *tauschii* (apart from 2 out of 56 accessions which were *strangulata*), whereas the majority of accessions in lineage 2 belonged to subspecies *strangulata*, except, nineteen accessions that had cylindrical spike morphology characteristic of ssp. *tauschii*. Lineage 1 covers accessions from Syria and USSR to Afghanistan and Pakistan, with majority of the accessions from eastern side of the Caspian region. For lineage 2, most of the accessions had origin in western region (Iran, Azerbaijan, Georgia), however, some accessions from USSR, Afghanistan, Turkmenistan, Tadzhikistan, and Kyrgyzstan were also clustered in lineage 2. The genetic relation between *Ae. tauschii* accessions was revealed by NJ tree constructed using genetic distances (**Figure 3B**). *Ae. tauschii* accessions were split into two widely distributed branches corresponding to lineage 1 and 2 with intermediate accessions between the two lineages. The results of all individual accessions were consistent between the NJ and the PCA.

To infer population structure, model based clustering was conducted using a set of 10,000 randomly selected polymorphic markers. The optimum number of clusters (*K*) was inferred to be two based on the structure plot. The accessions were assigned into two major clusters, referred as lineage 1 (red colored) and lineage 2 (blue colored) with some intermediate accessions represented as admixture (**Figure 3C**). The two major clusters inferred by *K* value are in correspondence with the division of *Ae. tauschii* accessions based on PCA and NJ analysis, lineage 1 composed of 56 accessions while lineage 2 has 114 accessions and seven intermediate accessions between the two lineages. The genetic differentiation among sub-lineages was tested using *F*-statistics and the pairwise *F*_ST_ was 0.56 between L1 and L2, supporting the lack of genetic flow between two lineages.

Grain size traits were compared in the two lineages and grain width and weight were found to differ significantly between the two lineages (Supplementary Figure S2 and Table S2). The mean grain weight of lineage 1 and lineage 2 was 0.60 and 0.77 g while mean grain width was 2.48 and 2.66 mm, respectively. The coefficient of variation was the highest for grain weight for both the lineages.

### Linkage Disequilibrium

**FIGURE 4:**
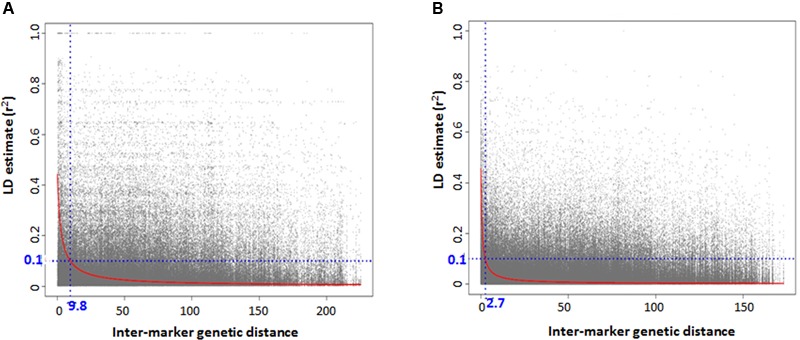
Genome-wide linkage disequilibrium (LD) decay plot for *Aegilops tauschii* accessions belonging to **(A)** lineage 1 and **(B)** lineage 2.

There was high LD for most of the pairwise comparisons between the SNP loci in *Ae. tauschii*. The presence of population structure was one reason inferred for high LD between independent loci and the LD analyses was then conducted separately for the two lineages. Based on the GBS data, accessions with high genetic similarity were identified and about 32% of accessions were discarded for LD analysis to avoid deviation in allele frequencies that is caused by presence of identical accessions. An association mapping panel consisting of 38 and 76 unique accessions in lineage 1 and 2, was thus selected. The extent of LD decay (*r*^2^ = 0.1) was found at an inter-marker genetic distance of 9.7 cM and 2.6 cM for lineage 1 and 2, respectively (**Figures 4A,B**). Two major factors that substantially reduced the amount of significant LD between independent marker pairs were removing very similar entries and taking into account the presence of population structure, however, there were around 15% of independent locus pairs that still showed significant LD in both the lineages individually which can be attributed to the presence of residual population structure within the lineages.

### Marker–Trait Associations for Grain Size and Weight

**FIGURE 5:**
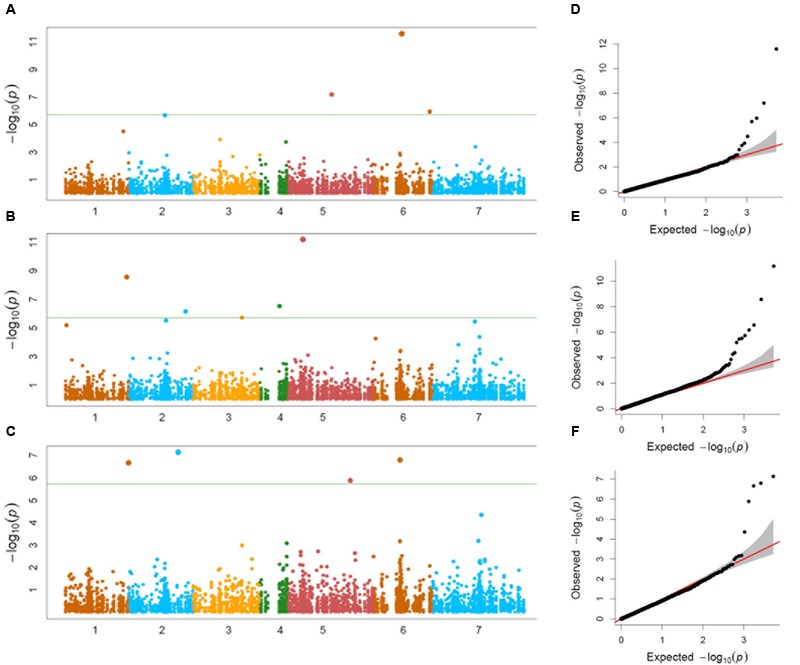
**(A–C)** Manhattan plots of **(A)** grain length, **(B)** grain width, and **(C)** grain weight for the seven chromosomes carrying the significant markers detected by MLM models using BLUP values; **(D–F)** Quantile–Quantile (Q–Q) plots for **(D)** grain length, **(E)** grain width, and **(F)** grain weight showing expected null distribution of *p*-values, assuming no associations, represented as solid red line; distribution of *p*-values observed.

Genome-wide association study conducted using a total of 114 *Ae. tauschii* accessions and 5,249 SNP markers detected a total of 17 MTAs above the threshold -log(P) score of 4.68, distributed on all the seven *Ae. tauschii* chromosomes (**Table 2**). Most significant associations for grain length, width and weight were detected for chromosomes 6D, 5D, and 2D at -log(P) scores of 11.6, 11.2, and 7.1, respectively (**Figures 5A–C**). The Q–Q plots illustrating observed associations between SNPs and grain length, width, and weight compared to expected associations after accounting for population structure and familial relatedness are depicted in **Figures 5D–F**.

**Table 2:** Most significant marker loci associated with grain length, width, and weight based on BLUP values of the *Ae. tauschii* data over 3 years 2011, 2012, and 2013.

Trait	SNP ID	Chromosome	Position (cM)^#^	*p*-Value	MAF	Effect^∗^	Putative candidate genes
Grain length	AT_27890	1D	143.5	3.20E-05	0.15	0.12	Myb-like DNA-binding domain
	AT_98099	2D	89.9	2.10E-06	0.12	0.10	Dual specificity phosphatase
	AT_14252	5D	109.4	6.70E-08	0.25	-0.16	–
	AT_68484	6D	71.1	2.60E-12	0.26	-0.17	3-ketoacyl-CoA synthase 6-like
	AT_29220	6D	140.0	1.20E-06	0.13	0.11	Cysteine-rich receptor-like protein kinase 6
Grain width	AT_85128	1D	151.9	2.70E-09	0.13	0.07	Putative serine/threonine-protein kinase receptor
	AT_16015	1D	3.4	6.60E-06	0.19	0.06	Cytochrome P450
	AT_67956	2D	141.0	7.20E-07	0.34	0.07	Pentatricopeptide repeat (PPR)-containing protein
	AT_96298	2D	92.5	3.10E-06	0.29	0.10	–
	AT_27226	3D	121.4	1.90E-06	0.25	-0.06	E3 ubiquitin-protein ligase TRIP12 isoform X6
	AT_104405	4D	49.1	2.90E-07	0.15	-0.06	germin-like protein 3 /oxalate oxidase
	AT_3134	5D	38.3	6.80E-12	0.39	0.08	Ethylene-insensitive protein 2
	AT_629	7D	105.0	3.70E-06	0.17	-0.05	Spastin (*Aegilops tauschii*)
Grain weight	AT_87410	1D	156.7	2.20E-07	0.30	0.03	Anaphase-promoting complex, subunit 10 (APC10)
	AT_8483	3D	121.4	1.00E-03	0.22	0.02	Auxin-induced protein 5NG4
	AT_95167	5D	155.4	1.40E-06	0.12	0.04	–
	AT_27138	6D	66.4	1.60E-07	0.27	-0.08	*S*-acyltransferase TIP1; DHHC palmitoyl transferase

*MAF, minor allele frequency.*

*^#^Position of the SNPs is according to POPSEQ data (Chapman et al., 2015; Edae et al., 2015).*

*^∗^An allelic effect size is the magnitude of the effect of an allele on a phenotype. Sign of the allelic effect estimate is with respect to the nucleotide that is second in alphabetical order. For associated SNP markers, where positive effects have been reported second allele in alphabetical order is favorable, and where negative effects have been reported, the first allele is favorable.*

**FIGURE 6:**
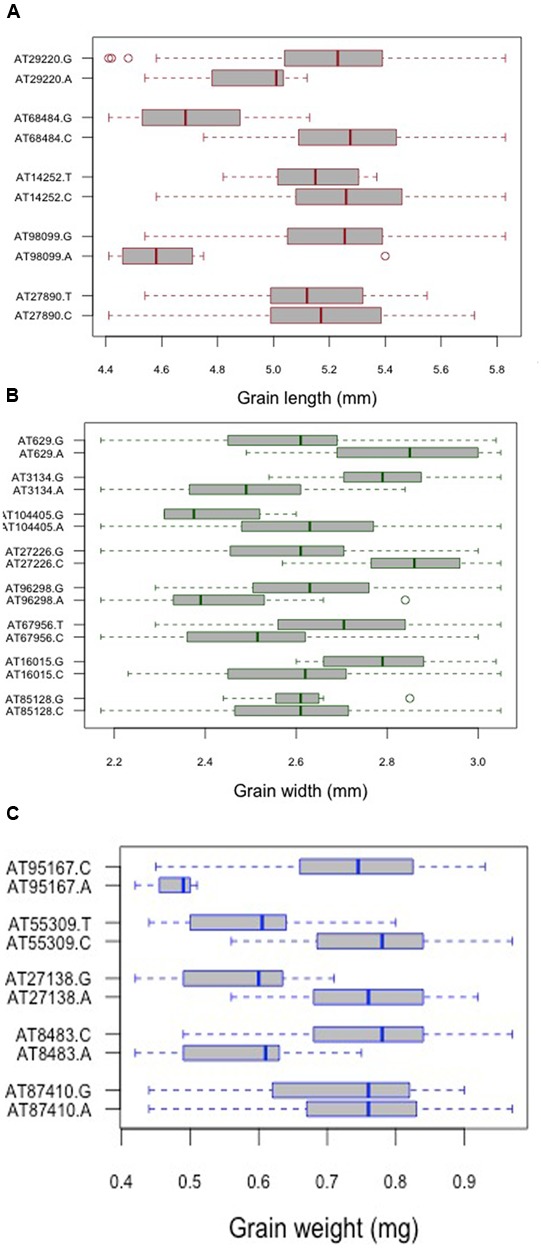
Comparison of the allelic effects for the SNP markers associated with **(A)** grain length, **(B)** grain width, and **(C)** grain weight. Kruskal–Wallis test was used to determine the significant differences between the mean values of two alleles.

For grain length, five associations, one each on chromosome 1D, 2D, 5D and two on 6D were mapped through GWAS with the most significant MTA on 6DS followed by that on 5D. For grain width, the most significant association was mapped on 5DS with a total of eight associations detected over the threshold value. For grain weight, four associations were mapped above the threshold -log(P) score (**Table 2**) with significant associations on chromosomes 2D, 6D, 1D, and 5D. Some of the chromosomal regions were detected to be associated with multiple grain architecture traits. A 4.7 cM (66.4–71.1 cM) region on 6D harbored two significant QTL, one each for grain length and grain weight. A 13.2 cM interval on 1D revealed associations with all the three traits grain length, width, and weight (**Table 2**). Grain length and width QTL on 2D were mapped at interval of 2.6 cM. Out of five marker associations for grain length, only one appeared to overlap with grain weight. For grain weight, however, significant associations on 5D was not detected at all in grain length and width indicating the contribution of genetic loci besides those controlling grain length and width. Grain weight associations on 1D and 6D were located on the chromosomal regions also harboring grain width and grain length associations, respectively albeit at a distance of more than 4.5 cM. The grain length, width, and weight were calculated for the two alleles of each of the associated SNP markers. The favorable alleles led to an increase varying from 0.58 to 10.9, 0.76 to 11.0, and 1.1 to 35.1% for grain length, width, and weight, respectively and variation is presented as box plots in **Figure 6**.

To find the relationship between the two lineages with respect to detected QTLs for grain weight and width, the mean values for each allele was compared between the lineages (Supplementary Table S3). It was found that there is significant difference in the mean values for both the alleles, with alleles in lineage 2 having higher mean values compared to lineage 1. This observation is supported by phenotypic analysis where it was reported that when grain traits were compared in the two lineages, both grain width and weight were found to differ significantly.

### Identification of Candidate Genes

SNPs tags for the linked SNPs were BLAST against TGACv1 chromosome sequences and chromosomal region spanning 10 kb on either side of the tag was used for gene prediction. For grain width, seven proteins were predicted from the sequences showing homology to linked SNPs and these included Putative serine/threonine-protein kinase receptors, cytochrome P450, pentatricopeptide repeat (PPR)-containing protein, E3 ubiquitin-protein ligase TRIP12 isoform X6, germin-like protein 3/oxalate oxidase, ethylene-insensitive protein 2 and Spastin protein (**Table 2**). For grain length, proteins were predicted for four associated SNPs with Myb-like DNA-binding domain, dual specificity phosphatase, 3-ketoacyl-CoA synthase 6-like, and cysteine-rich receptor-like protein kinase 6. For grain weight, MTA on chromosome 1D and 6D were predicted as anaphase-promoting complex, subunit 10 (APC10) and *S*-acyltransferase TIP1; DHHC palmitoyl transferase, respectively.

## Discussion

The main objective of the present investigation was to mine the grain size variability encompassed in *Ae. tauschii*, the D-genome donor of hexaploid wheat, and to identify the genomic regions controlling the grain architecture.

### Phenotypic Evaluation

In this study, diverse *Ae. tauschii* accessions showed significant level of variation for grain size and weight with moderate heritability. Significant positive correlation of grain length and width with grain weight indicated that increase in grain length and width both contribute to enhanced grain weight. However, grain width comparatively had more positive impact on grain weight than grain length in the present study. Moderate to strong correlations between grain weight and size have been reported (Rasheed et al., 2014) in other studies. Simmonds et al. (2016) observed that both grain width and length, in tetraploid and hexaploid wheat, led to the increase in thousand grain weight due to a mutation in *TaGW2-A1*. Correlations between these traits points to a causal relationship between grain size and weight as longer and broader grains are able to accumulate more starch and hence have more grain weight.

### Molecular Diversity and Structure Analysis

*Aegilops tauschii* has a wide geographic distribution and tremendous natural variation for agronomically important genes. Genetic variations for flowering time and several other morphological traits have been reported (Mansouri et al., 2013). *Ae. tauschii* genetic diversity has also been studied using SSR (Pestsova et al., 2000), AFLP (Mizuno et al., 2010), and SNP (Wang et al., 2013) markers. We used GBS-based SNP markers to understand the molecular diversity in *Ae. tauschii*. Based on phylogenetic tree, PCA and structure analysis, *Ae. tauschii* accessions were divided into two lineages (L1 and L2) in concordance with previous studies (Mansouri et al., 2013). Substantial phenotypic differences were observed for grain width and weight between these two lineages. Grains in lineage 2 were on average heavier and wider, whereas, those in lineage 1 were comparatively lighter and thinner. Most of the lineage 2 accessions belonged to ssp. *strangulata* with monliform spikes and lineage 1 had accessions with cylindrical spikes from ssp. *tauschii*. In previous studies conducted by Mansouri et al. (2013) differences in grain weight between the two lineages of *Ae. tauschii* were recorded with higher grain weight for ssp. *strangulata* accessions.

GBS marker-based division of *Ae. tauschii* panel into two lineages did not correspond well to the absolute differentiation based on two subspecies (*strangulata* and *tauschii*). The majority of lineage 2 accessions belonged to ssp. *strangulata*, however, we found nineteen accessions that had cylindrical spike morphology characteristic of ssp. *tauschii* which might have resulted due to recent hybridizations between two sub species. Some previous reports have also revealed grouping of ssp. *tauschii* accessions with those of ssp. *strangulata* (Mizuno et al., 2010). In addition, some accessions were intermediate between the two lineages. The presence of intermediate accessions was also reported by Pestsova et al. (2000), Mizuno et al. (2010), and Wang et al. (2013). Mizuno et al. (2010) referred to these intermediates as separate cluster and suggested that it might be one of the ancestral lineage of *Ae. tauschii* from which the two lineages diverged or it may have happened due to chance hybridizations between the two subspecies (Lubbers et al., 1991). Both the lineages were further subdivided into sub-lineages, however, we could not find absolute geographic isolations within sub-lineages which is contradictory to other studies that reported complete geographic isolation of sub-lineages into east and west habitats (Mizuno et al., 2010; Wang et al., 2013).

Accessions with high level of genetic similarities assessed based on GBS-SNP markers and phenotypic similarities were removed from the association mapping panel which even though led to the reduction in the panel size but took care of the potentially inflated allele frequencies and LD among unlinked loci, which might have led to false associations. LD analysis was conducted separately in both lineages but still there were around 15% of independent locus pairs that showed significant LD in both the lineages individually. It can be attributed either to residual population structure existing within lineages or selection or recent hybridizations between the two lineages. There was difference in LD between the lineages. Lineage 1 has more extensive LD with *r*^2^ = 0.1 at inter-marker genetic distance of 9.7 cM compared to 2.6 cM LD in lineage 2. One possible explanation for longer LD observed in lineage 1 is fewer number of recombination events in its evolutionary history compared to lineage 2 or due to smaller population size. Previous diversity analysis studies have established that lineage 2 was more diverse than lineage 1 and lineage diversification occurred by west to east dispersal of accessions (Mizuno et al., 2010; Wang et al., 2013). Another cause could be relatively small number of accessions in lineage 1, which can lead to biased estimates of LD.

### Marker–Trait Associations

GWAS for grain shape and weight was conducted for an association mapping panel consisting of 114 *Ae. tauschii* accessions belonging to both the lineages. First three principal components were used as covariates to take into account the underlying population structure during GWAS. Association mapping panels having strong structure can be used for GWAS after taking into consideration the population structure as has been demonstrated in rice (McCouch et al., 2016). Overall, 17 MTAs were identified on all the seven *Ae. tauschii* chromosomes. A large number of studies have reported mapping for grain size and weight in hexaploid wheat but limited studies are available where grain size and grain weight variation has been elucidated in *Ae. tauschii* itself. To the best of our knowledge only a single study has reported mapping these traits in *Ae. tauschii* germplasm using GWAS (Zhao et al., 2015). Okamoto et al. (2013) and Rasheed et al. (2014) mapped grain size and shape QTLs on D-genome in synthetic wheats using biparental mapping populations and GWAS, respectively. Comparison of the chromosomal regions, however, could not be done as studies cited earlier have either used SSR or DArT markers. SNP marker loci location in the present study were based on the PopSeq map (Edae et al., 2015).

Most significant QTLs for grain length, width, and weight were mapped on different chromosomes, viz., 6D, 5D, and 2D, respectively. However, some of the chromosomal regions such as 6D_66.4–71.1 cM, 1D_143.5–156.7 cM, and 2D_89.9–92.5 cM had QTL for multiple grain traits. Grain length QTL on 5D and 6D in *Ae. tauschii* were also observed by Zhao et al. (2015) and on 2D, 4D, and 7D by Okamoto et al. (2013) in synthetic wheat based populations. We, however, did not map any significant QTL on 4D and 7D. Grain width was observed to be significantly associated with more SNPs than the other traits, viz., eight SNPs over six chromosomes. Chromosome 4D and 7D were earlier reported to be associated with grain width by Cui et al. (2011) in their study on wheat RIL populations. Only chromosome 1D had QTL for all the three traits. One of the grain length QTL region on 6D also contributed to grain weight. None of the other grain length and/or length QTL showed overlap with grain weight QTL. Most significant QTL for grain weight were independent from grain length and width indicating factors other than length and width playing significant role in grain weight. QTLs for grain weight were reported in synthetic wheat populations on chromosomes 1D and 5D (Okamoto et al., 2013) and wheat mapping populations on chromosome 2D (Cuthbert et al., 2008) and 7D (Groos et al., 2003; Huang et al., 2003; Mir et al., 2012). We also detected MTAs on chromosome 1D and 5D but no significant MTAs were seen on 2D and 7D.

Limited understanding of grain size and shape is available in polyploid wheat where QTL for grain size and shape have been identified (Zhang et al., 2010; Williams and Sorrells, 2014; Simmonds et al., 2016), but no gene affecting grain weight independent of length and width has yet been cloned. This is in contrast to rice where genes with large effects on grain size have been identified revealing an independent genetic control of grain length and width (Weng et al., 2008). In wheat though *TaGW2* has been shown to be a negative regulator of grain weight as mutant alleles of *TaGW2-A1* have been found to increase grain weight by contributing both to length and width (Simmonds et al., 2016). Genes controlling seed size have been extensively studied in rice. The characterized genes have been revealed to function in G-protein signaling (Huang et al., 2009), or in the ubiquitin–proteasome pathway (Song et al., 2007). *TaGS5* an ortholog of rice *GS5* has been shown to be a positive regulator for grain size in wheat (Ma et al., 2016). In rice, a gene *GL3.1*, regulating grain length and yield was cloned and found to belong to serine/threonine phosphatase of the PPKL family. *GL3.1* regulates grain length by mediating cell cycle progression through affecting the phosphorylation status of cell cycle proteins, such as cyclin-T1;3, thereby controlling grain yield (Qi et al., 2012).

In the present study, transcription factors that regulate cell proliferation and differentiation with Myb-like DNA-binding domain were predicted for SNPs associated with grain length. Grain length associated SNP marker AT_98099-2D was observed to lie in the genomic regions harboring dual (serine/threonine and tyrosine)-specific phosphatases in the present investigation as well. For grain width, most significant QTL on 5D was found to be an ethylene insensitive protein 2 which has been reported to play a central role in signaling pathways regulated by ethylene and involved in various processes including development, plant defense, senescence, and nucleotide sugar flux. Reduction in ethylene signaling has been proposed to increase grain yield (Shi et al., 2015). Li et al. (2016) demonstrated that overexpression of wheat gene PPR protein designated TaMRRP (*TaMOR*) improved root system architecture and resulted in higher grain yield in rice and in the present investigation SNP marker AT_67956_2D was found to be in or around a PPR protein.

The ubiquitin pathway has been known to play an important part in plant seed size determination (Li and Li, 2014) and rice gene *GW2* has been found to encode a RING-type E3 ubiquitin ligase and is a negative regulator of grain width and weight (Matsuoka and Ashikari, 2007). Wheat also has orthologous copies of *GW2* on 6A, 6B, and 6D with A genome copy being most studied. In the present investigation, however, E3 ubiquitin-protein ligase TRIP12 isoform X6 was predicted for the MTA on 3D for grain width. Germin-like protein 3-oxalate oxidase predicted for grain width associated SNP locus AT_104405_4D have been implicated in a variety of plant processes including germination, development, pollen formation, and response to abiotic and biotic stress (Davidson et al., 2009). Another grain width associated SNP AT_85128 was observed to be located on the *Ae. tauschii* genomic region harboring putative serine/threonine-protein kinase receptor. For the grain weight associated SNPs, major predicted proteins belonged to the class of APC10 which is an important conserved multi-subunit ubiquitin ligase, marking targets for degradation by the 26S proteasome (Heyman and De Veylder, 2012).

High grain size *Ae. tauschii* accessions identified in the present study and associated markers constitute an important genetic and genomic resource with a potential to contribute important genetic variability for the wheat gene pool for grain size and weight. Wheat being a hexaploid has immense buffering capacity and how *Ae. tauschii* alleles for grain weight/grain size express in the hexaploid wheat background still needs to be investigated? Although a number of other traits including disease resistance and quality traits have been transferred from *Ae. tauschii* to cultivated wheat by various groups. SNP markers linked to the grain architecture attributes, however, can be used for validation and marker assisted introgression of these traits in cultivated wheat background either using *Triticum durum* as bridging species or in direct crosses with hexaploid wheat.

## Conclusion

The present work was conducted to understand the genetic and phenotypic diversity in a collection of *Ae. tauschii* accessions and unravel the genetic architecture of grain size by exploring the historical recombination using LD mapping. *Ae. tauschii* had large variation for grain size and strong population structure that divides it into two lineages. The mean values for both grain width and weight differed significantly between the lineages. Gaining insight into both genetic and morphological diversity will help to make selection for the accessions, which are both morphologically and genetically diverse. GBS is a cost-effective tool to make the collections non-redundant and also saves time and money spent on phenotyping of identical accessions. *Ae. tauschii* chromosomes recombine easily with the D-genome chromosomes of bread wheat and can be exploited easily using *T. durum* as a bridging species. The mapping of favorable alleles gives the opportunities to incorporate the alleles that were excluded from the domesticated gene pool as a result of domestication processes. Further investigations need to be conducted to confirm the relative importance of the chromosomal regions/genes associated with grain size.

## Author Contributions

SA carried out the phenotyping of the germplasm, analyzed both genotype and phenotype data, and wrote the draft of the manuscript; NS helped in the SNP detection and diversity analysis; SK maintained the germplasm in the field and helped in the phenotyping; NB helped in the selection of the panel, analysis, and interpretation of the results; CU helped in genome-wide association mapping, manuscript preparation, and candidate gene search; JP supervised genotyping by sequencing of the *Ae. tauschii* mapping panel, diversity analysis, genome-wide association mapping, and helped in manuscript preparation. PC conceived the idea, designed and supervised the study, prepared the draft of the manuscript, and submitted it. All the authors have read the manuscript and approve it.

## Conflict of Interest Statement

The authors declare that the research was conducted in the absence of any commercial or financial relationships that could be construed as a potential conflict of interest.

## Footnotes

1 http://plants.ensembl.org/

2 http://www.ncbi.nlm.nih.gov/

## Supplementary Material

TABLE S1 The list of the Ae. tauschii accessions used in this study, their origin, lineage and spike type.
