# Supplementary material for: Genome-Wide Association Study of Grain Architecture in Wild Wheat Aegilops tauschii
Source: Front Plant Sci. 2017 May 31;8:886. doi: 10.3389/fpls.2017.00886 (PMC5450224; doi:10.3389/fpls.2017.00886)
Supplement: TABLE S1 — The list of the Ae. tauschii accessions used in this study, their origin, lineage and spike type. [file Presentation_1.pdf]

Supplementary Information

Genome-Wide Association Study of Grain Architecture in Wild Wheat *Aegilops tauschii*

Sanu Arora[1,3], Narinder Singh[2], Satinder Kaur[1], Navtej Singh Bains[1], Cristobal Uauy[3], Jesse Poland[2], Parveen Chhuneja*[1]

[1] School of Agricultural Biotechnology, Punjab Agricultural University, Ludhiana, Punjab, 141004, India

[2] Wheat Genetics Resource Center, Department of Plant Pathology, Kansas State University, Manhattan, KS, 66506, USA

[3] Crop Genetics, John Innes Centre, Norwich Research Park, NR4 7UH, UK

[*] Corresponding Author – Parveen Chhuneja, Email: pchhuneja@pau.edu Fax: +91-161-2401444

PAU_Accession_No.	Original_Accession_No.	Origin	Lineage	Spike_type
pau13	79 TDO 6-3	Unknown	L2	S
pau17	79 TD 18-3	Unknown	L2	S
pau3544	349037	USSR	L2	S
pau3733	-	USSR	L1	T
pau3735	PI431600	USSR	L1	M
pau3742	O 2016/Clae2	Pakistan	L1	T
pau3744	O 2038	Afghanistan	L1	T
pau3746	O 2095/Clae6	Afghanistan	L2	T
pau3750	O 2118/Clae11	Iran	L2	S
pau3751	O 2119/Clae12	Iran	L2	S
pau3752	O 2123/Clae13	Iran	L2	S
pau3753	O2128/Clae14	Iran	L1	T
pau3755	O 2133	Iran	L2	S
pau3757	O 2137/Clae18	Iran	L2	S
pau3758	O 2139/Clae19	Iran	L2	T
pau3759	O2140	Unknown	L1	T
pau3760	O 2141	Iran	L2	S
pau3761	O 2142	Iran	L2	S
pau3766	O 2168/Clae27	Iran	L1	T
pau3769	O 2402	Unknown	L2	S
pau3784	O SANDO	Unknown	L2	T
pau3798	-	Unknown	L2	S
pau3799	-	Unknown	L2	S
pau3805	O M7-262	Iran	L2	S
pau3806	0 0-623	Unknown	L1	T
pau3823	TQ 08	Iran	L2	S
pau3826	RL 5271	Unknown	L1	S
pau9785	TA1653	Tajikistan	L1	T
pau9787	TA1661	Azerbaijan	L2	S
pau9788	TA1664	Azerbaijan	L2	S
pau9790	TA1669	Azerbaijan	L2	S
pau9791	TA1670	Azerbaijan	L1	T
pau9795	TA1677	Azerbaijan	L1	T
pau9796	TA1686	Azerbaijan	L2	S
pau9798	TA1691	Japan	L2	S
pau9800	TA1694	Turkmenistan	L2	S
pau9804	TA 1717	Iran	L2	T
pau9806	TA 2458	Iran	L2	S
pau9807	TA 2460	Iran	L2	S
pau9809	TA2468	Iran	L2	S
pau9822	TA 2380	Pakistan	L1	T
pau9823	TA 2385	Pakistan	L2	T
pau9824	TA 2390	Afghanistan	L1	T
pau9829	TA 2419	Afghanistan	L1	T

pau9830	TA 2420	Afghanistan	L1	T
pau13757	IG312287	Unknown	L1	T
pau13761	312291	Unknown	L2	M
pau13762	IG312292	Unknown	L1	T
pau13765	IG312295	Unknown	L1	T
pau13780	-	Unknown	L1	T
pau13781	-	Unknown	L1	T
pau14096	AE145	Azerbaijan	L2	S
pau14100	AE149	Unknown	L1	T
pau14102	AE179	Azerbaijan	L2	T
pau14103	AE181	Azerbaijan	L1	S
pau14104	AE183	Iran	L1	T
pau14105	AE184	Iran	L1	T
pau14106	AE188	Turkeminstan	L1	T
pau14109	AE191	Azerbaijan	L2	S
pau14111	AE193	Afghanistan	L1	T
pau14113	AE195	Azerbaijan	L2	S
pau14115	AE197	Azerbaijan	L2	S
pau14116	AE198	Azerbaijan	L2	S
pau14118	AE200	Azerbaijan	L2	S
pau14122	AE204	Azerbaijan	L2	S
pau14128	AE210	Azerbaijan	L2	S
pau14129	AE211	Azerbaijan	L2	S
pau14130	AE212	Turkmenistan	L2	S
pau14135	AE217	Azerbaijan	L2	S
pau14136	AE218	Azerbaijan	L2	S
pau14138	AE220	Azerbaijan	L2	S
pau14139	AE221	Azerbaijan	L2	S
pau14140	AE222	Azerbaijan	L2	S
pau14145	AE228	Azerbaijan	L2	S
pau14147	AE230	Azerbaijan	L2	S
pau14158	AE241	Uzbekistan	L2	S
pau14159	AE242	Turkmenistan	L2	T
pau14160	AE245	Armenia	L2	T
pau14163	AE248	Turkeminstan	L1	T
pau14165	AE250	Turkmenistan	L2	S
pau14166	AE251	Azerbaijan	L2	T
pau14170	AE256	Kyrgyzstan	L2	S
pau14174	AE261	Azerbaijan	L1	T
pau14175	AE262	Azerbaijan	L2	S
pau14177	AE264	Azerbaijan	L1	T
pau14180	AE267	Azerbaijan	L2	S
pau14181	AE268	Azerbaijan	L2	S
pau14185	AE273	Azerbaijan	L2	S
pau14186	AE275	Afghanistan	L2	S

pau14187	AE276	Afghanistan	L2	S
pau14190	AE279	Afghanistan	L2	S
pau14194	AE291	Turkmenistan	L2	S
pau14197	AE422	Unknown	L1	T
pau14200	AE425	Unknown	L2	S
pau14201	AE426	Unknown	L2	S
pau14203	AE428	Unknown	L2	M
pau14204	AE429	Unknown	L2	S
pau14205	AE430	Unknown	L2	T
pau14206	AE431	Unknown	L2	T
pau14209	AE434	Unknown	L2	T
pau14210	AE454	Georgia	L2	S
pau14211	AE457	Georgia	L2	S
pau14214	AE472	Turkeminstan	L1	T
pau14217	AE490	Soveit Union	L2	T
pau14223	AE541	Iran	L2	T
pau14227	AE636	Turkmenistan	L2	S
pau14228	AE637	Turkeminstan	L1	T
pau14229	AE639	Soveit Union	L1	M
pau14230	AE647	Tajikistan	L1	T
pau14231	AE692	Soveit Union	L2	S
pau14232	AE721	Armenia	L2	T
pau14236	AE817	Tajikistan	L1	T
pau14237	AE841	Turkmenistan	L2	T
pau14238	AE858	Tadzhikistan	L2	T
pau14240	AE933	Georgia	L2	S
pau14241	AE938	Armenia	L2	S
pau14242	AE940	Armenia	L2	T
pau14246	AE964	Turkmenistan	L2	S
pau14251	AE1055	Azerbaijan	L1	T
pau14253	AE1069	Syria	L1	T
pau14254	AE1087	Afghanistan	L1	T
pau14323	7-2	Unknown	L2	S
pau14325	11-1	Unknown	L1	T
pau14330	16-4	Unknown	L2	S
pau14334	20-2	Unknown	L2	S
pau14336	21-3	Unknown	L2	S
pau14337	21-4	Unknown	L2	S
pau14338	22-2	Unknown	L2	S
pau14339	22-3	Unknown	L2	S
pau14340	22-4	Unknown	L2	S
pau14341	24-4	Unknown	L2	S
pau14343	27-2	Unknown	L2	S
pau14345	28-2	Unknown	L2	S
pau14347	28-4	Unknown	L2	M

pau14348	32-1	Unknown	L2	S
pau14351	34-2	Unknown	L1	T
pau14352	34-3	Unknown	L1	T
pau14353	34-4	Unknown	L1	T
pau14354	35-1	Unknown	L1	T
pau14355	36-4	Unknown	L1	T
pau14356	37-3	Unknown	L2	S
pau14359	40-1	Unknown	L2	S
pau14360	40-2	Unknown	L2	S
pau14362	40-4	Unknown	L2	S
pau14576	30-2	Unknown	L2	S
pau14578	89809-A	Unknown	L1	T
pau14582	23-4	Unknown	L2	S
pau14583	26-2	Unknown	L2	S
pau14586	30-4	Unknown	L2	T
pau14953	AE061D	Pakistan	L1	T
pau14954	Clae-8	Iran	L2	S
pau14957	Clae-17	Iran	L2	S
pau14958	Clae-18	Iran	L2	S
pau14960	Clae24	Iran	L2	S
pau14962	AE061D	Iran	L1	T
pau14966	AE091D	Unknown	L1	T
pau14967	Clae72	Unknown	L2	S
pau14968	AE061D	Afghanistan	L1	T
pau14970	AE061D	Unknown	L1	T
pau14972	PI-349037	Azerbaijan	L2	S
pau14973	PI-369627	Unknown	L2	S
pau14974	PI-428563	Georgia	L2	S
pau14975	AE061D	Turkmenistan	L1	T
pau14979	PI 486270	Turkey	L1	T
pau14985	AE951D	Unknown	L1	M
pau14990	PI 560756	Turkey	L1	T
pau14992	TA1651	Iran	L2	S
pau14995	TA1695	Japan	L2	S
pau14998	PI 603250	Iran	L2	S
pau14999	AE991D	Iran	L1	T
pau13764*	IG312294	Unknown	Intermediate	T
pau14088*	-	Soviet Union	Intermediate	T
pau14091*	AE67	Unknown	Intermediate	S
pau14092*	AE141	Turkeminstan	Intermediate	T
pau14156*	AE239	Uzbekistan	Intermediate	S
pau14162*	AE247	Turkeminstan	Intermediate	T
pau14195*	AE398	Turkeminstan	Intermediate	T

Table S2: Descriptive statistics for grain size descriptors of two *Ae. tauschii* lineages (L1, L2) for grain length; grain width and grain weight

Trait	Lineage 1 (L1)			Lineage 2 (L2)		
	**Mean**	**S.D.**	**C.V%**	**Mean**	**S.D.**	**C.V%**
Grain length (mm)	5.15	0.31	6.00	5.18	0.37	7.00
Grain width (mm)	2.48	0.20	8.00	2.66	0.19	7.00
Grain weight (mg)	0.60	0.12	20.00	0.77	0.10	14.00

Table S3: Mean grain weight and width for each allele of the associated SNP in both lineages

Trait	SNP ID	Allele 1-L1	Allele 2 – L1	Allele 1 –L2	Allele 2 – L2
Grain Weight	AT_87410	A = 0.55	G = 0.62	A = 0.77	G = 0.82
	AT_8483	A = 0.55	C = 0.61	A = X	C = 0.78
	AT_95167	A = 0.47	C = 0.62	A = X	C = 0.77
	AT_27138	A = 0.56	G = 0.67	A = 0.76	G = X
Grain Width	AT_85128	C = 2.41	G = 2.58	C = 2.64	G = 2.85
	AT_16015	C = 2.36	G = 2.72	C = 2.65	G = 2.85
	AT_67956	C = 2.41	T = X	C = 2.54	T = 2.70
	AT_96298	A = 2.41	G = X	A = X	G = 2.64
	AT_27226	C = 2.42	G = X	C = 2.85	G = 2.61
	AT_104405	A = 2.45	G = 2.41	A = 2.69	G = 2.57
	AT_3134	A = 2.42	G = X	A = 2.53	G = 2.79
	AT_629	G = 2.45	A = X	G = 2.61	A = 2.81

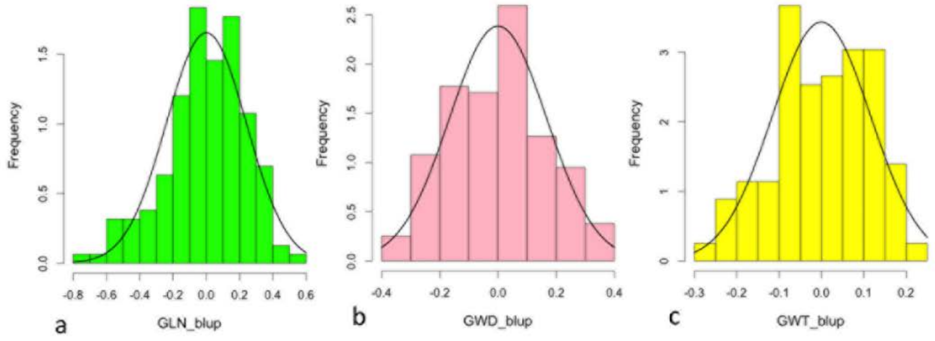

Supplementary Figure S1. Distribution of best linear unbiased predictions (BLUP) values with normality curve for grain (a) grain length (b) grain width and (c) grain weight.

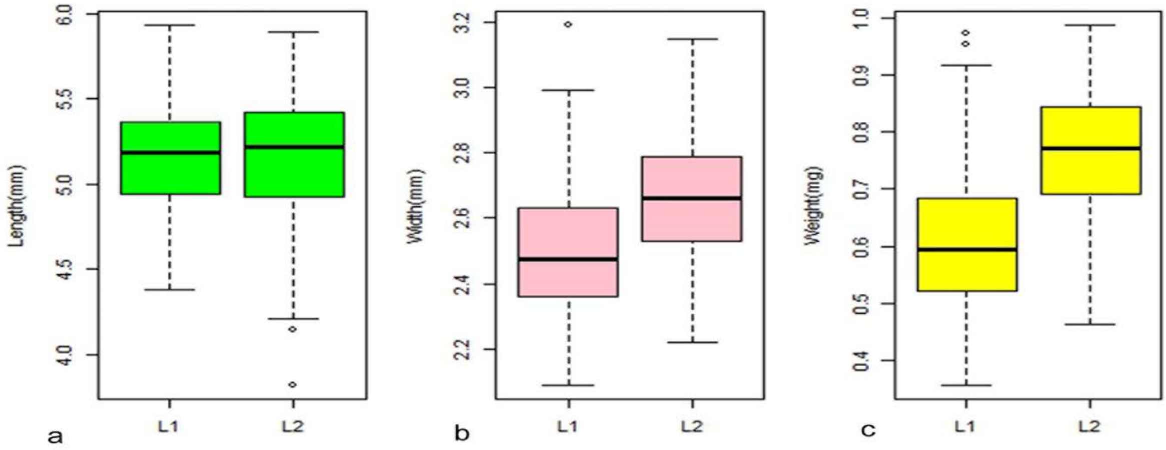

Supplementary Figure S2: Boxplots showing mean, median and range of phenotypic variation for the two lineages of *Ae. tauschii* for (a) grain length (mm) (b) grain width (mm) and (c) grain weight (mg).
